# Process evaluation of the Hockey Fans in Training lifestyle intervention (for men with overweight or obesity)

**DOI:** 10.1093/tbm/ibaf002

**Published:** 2025-02-18

**Authors:** Wendy M Blunt, Marisa L Kfrerer, Dawn P Gill, Katie J Shillington, Brendan Riggin, Jennifer D Irwin, Brooke Bliss, Robert J Petrella

**Affiliations:** Centre for Studies in Family Medicine, Department of Family Medicine, Schulich School of Medicine and Dentistry, Western University, London, Ontario, N6G 2M1, Canada; Centre for Studies in Family Medicine, Department of Family Medicine, Schulich School of Medicine and Dentistry, Western University, London, Ontario, N6G 2M1, Canada; Centre for Studies in Family Medicine, Department of Family Medicine, Schulich School of Medicine and Dentistry, Western University, London, Ontario, N6G 2M1, Canada; Health and Rehabilitation Sciences, Faculty of Health Sciences, The University of Western Ontario, London, Ontario, N6A 3K7, Canada; Department of Neurobiology, University of California San Diego, La Jolla, CA 92093, USA; Center for Empathy and Social Justice in Human Health, T. Denny Sanford Institute for Empathy and Compassion, University of California San Diego, La Jolla, CA 92093, USA; Centre for Studies in Family Medicine, Department of Family Medicine, Schulich School of Medicine and Dentistry, Western University, London, Ontario, N6G 2M1, Canada; Department of Recreation and Leisure Studies, Faculty of Heath, University of Waterloo, Waterloo, Ontario, N2L 3G1, Canada; Health and Rehabilitation Sciences, Faculty of Health Sciences, The University of Western Ontario, London, Ontario, N6A 3K7, Canada; School of Health Studies, Faculty of Health Sciences, The University of Western Ontario, London, Ontario, Canada; Centre for Studies in Family Medicine, Department of Family Medicine, Schulich School of Medicine and Dentistry, Western University, London, Ontario, N6G 2M1, Canada; Centre for Studies in Family Medicine, Department of Family Medicine, Schulich School of Medicine and Dentistry, Western University, London, Ontario, N6G 2M1, Canada; Department of Family Practice, Faculty of Medicine, University of British Columbia, Vancouver, British Columbia, Canada; The Bone and Joint Institute, The University of Western Ontario, London, Ontario, N6A 3K7, Canada; School of Kinesiology, Faculty of Education, University of British Columbia, Vancouver, British Columbia, V6T 1Z1, Canada

**Keywords:** overweight, obesity, men, sport fandom, health behaviour, process evaluation

## Abstract

**Background:**

Despite the proven relationship between lifestyle and morbidity and mortality, rates of chronic disease (e.g. obesity) continue to rise in paradox to the myriad of studies supporting lifestyle behaviour change. Men have been less likely to seek out preventative care or lifestyle programs, putting them at risk. In response, Hockey Fans In Training (Hockey FIT) was developed as a group-based, lifestyle intervention leveraging the draw of hockey fandom to engage middle-aged men with overweight or obesity in lifestyle change. Encouraging pilot study results informed the optimization and delivery of the intervention through a cluster randomized controlled trial in 42 sites in Canada and the USA.

**Methods:**

A process evaluation was conducted to evaluate intervention acceptability and fidelity and adaptations. Community-based sites were randomly allocated to Hockey FIT intervention (immediate program start) or wait-list control (12-month delay). Qualitative process evaluation data were collected from intervention sites and included seven virtual participant focus groups and one interview (*n* = 35 participants), open-ended participant feedback questionnaires (*n* = 316), interviews with program coaches (*n* = 22), post-session coach reflections (*n* = 233), and interviews with implementation partners (*n* = 16). A process of content analysis by question was performed and data saturation was reached.

**Results:**

Themes fell into the following categories: (i) motivations for joining Hockey FIT; (ii) effective program components; and (iii) adaptations and suggested improvements.

**Conclusions:**

The process evaluation detailed success engaging men in lifestyle change using sport fandom, and the importance of capitalizing further on competition within groups to drive behavioural change through user-friendly supports and greater engagement with hockey.

**Clinical Trial information:**

ClinicalTrials.gov. ID: NCT03636282

## Teaser

Group dynamics in pursuit of cohesiveness are a pivotal part in creating health behaviour change. Working within groups or communities with similar interests (such as hockey fans) could be a strong foundation for creating and maintaining behaviour change.

**Implications**

Practice: Bringing together communities and partners with similar interests in sport can be an effective method for implementing interventions focused on health behaviour changes to reduce chronic disease risk.

Policy: Policymakers should support communities with common interests in sport to implement programs to address growing rates of chronic disease in Canada.

Research: Future research should focus on expanding the success of programs like Hockey FIT to additional communities with similar interests (e.g. other sports, population groups, and hobbies).

## Introduction

Overweight or obesity and associated risk factors, such as physical inactivity and unhealthy diet, may lead to a number of preventable chronic diseases including diabetes, stroke, cancer, heart disease, and depression [1–3]. In 2018, 26.8% of Canadians had obesity, representing one in four Canadian adults [1]. This is a notable increase from 2014 wherein 20.2% of Canadian adults reported a weight-to-height ratio indicating obesity [4]. In the USA, the burden is greater, with 42.4% having obesity [5]. For Canadian adults, only 53% self-report reaching the recommended 150 minutes of exercise each week, 22% are consuming five or more servings of fruit/vegetables per day, and 71% are meeting or exceeding the threshold for sedentary time per day [6–8]. Further, there is a worrisome trend of men consuming less fruits and vegetables, and accessing less services to promote health, leaving them at a possible increased risk for chronic disease compared to other genders [9].

Given that chronic disease rates are projected to rise over the next two decades, with a large proportion being in adult men, there is a need for interventions and research contextualizing the results of gender-sensitized interventions to scale-up and tailor appropriately to men to promote health [9]. Recent literature suggests a complex relationship when it comes to the impact of masculinity on men’s health behaviours [10]. This finding highlights the need for programs to work within preferences in health promotion and health behaviour change to engage men who are harder to reach with preventative programs. The practice of masculinity posits self-reliance and independence as core values to expressing a masculine identity [11]. These values in practice may impact men’s engagement in health-seeking behaviour. For instance, when it comes to preventative care, men may be less likely to seek health care until their independence is affected, which tends to be later on in the process of disease progression [12]. This delay may reduce engagement in activities to prevent chronic disease [13]. Men may also be more likely to view lifestyle interventions as inherently feminine, leading to challenges recruiting men in preventative care [14]. Subsequently, implementing programs that attend to masculine narratives to endorse health-promoting behaviours, is key to improving men’s health at large.

Researchers are increasingly turning towards sport to promote healthy lifestyles, using professional sport team fandom to target men’s values and interests [15, 16]. Previous studies have reported high engagement by leveraging sport fandom to promote healthy lifestyle changes in men [15, 16]. Watching sports is often an enjoyable experience with family and friends [17]. Sport fandom can be seen as a key component of identity and feelings of inclusiveness within a broader community [18]. There is also a paradox that exists with health and sport fanship. Researchers have found sports fans feel a need to choose between their physical health and well-being, and their identity as a sports fan [19]. Therefore, promising interventions to improve health in men ought to attend to both health and sport fanship.

The sport of hockey has been identified as an integral part of North American identity, with over two-thirds of Canadians and 13 million Americans identifying as a hockey fan [20–22]. To leverage this strong connection to the sport of hockey, Hockey Fans In Training (Hockey FIT) was created and piloted using major junior or professional hockey teams to engage at-risk men in changing and sustaining healthy lifestyle behaviours [23–25]. Hockey FIT was developed, adapted, and optimized from a smaller-scale pilot randomized controlled trial (RCT) and informed by other lifestyle interventions, including those that utilized sport fandom to engage men [16, 26, 27]. In brief, the pilot proved successful in promoting health behavioural change and weight loss amongst participants, which were maintained up to 1 year post-intervention [25]. A process evaluation was conducted as part of the pilot project to inform the adaptation and implementation of the intervention within a full-scale effectiveness trial [23].

The aim of this paper is to report on the process evaluation of Hockey FIT conducted alongside the full-scale pragmatic, cluster RCT. Specifically, this process evaluation sought to explore the acceptability of the Hockey FIT program, and where the program can improve to better meet coach, partner, and participant needs. The objectives were to explore:

1. *Program acceptability*: To what extent did participants, coaches, and partners find the program appropriate for encouraging health behaviour change?
2. *Fidelity and adaptations*: To what extent was the program delivered as designed? What adaptations were made or suggested to be made to improve the program in the future?

## Materials and Methods

The research team’s approach to data analysis was through an interpretivist lens. Interpretivism is focused on contextualizing and understanding the social reality of human experiences [28]. Thus, the research team sought to understand multiple realities from those involved in Hockey FIT to contextualize program acceptability, and fidelity and adaptations [29].

### Study design

The Hockey FIT pragmatic, cluster RCT design, rationale, procedures, and results have been described in detail elsewhere [30, 31]. The overall purpose of this larger cluster RCT was to evaluate effectiveness, cost-effectiveness, and implementation of Hockey FIT in multiple sites across Canada and the USA, the latter of which this paper reports. The use of the cluster design was to minimize experimental contamination across members of the cluster as the pilot Hockey FIT trial revealed a higher potential for contamination when allocated within site [25]. The Hockey FIT intervention ran from January 2019 through to April 2023 with staggered starts based on regional proximity for logistics purposes and site readiness to participate. Stratum 1 (sites in Ontario, Canada) began implementation in January 2019, and Stratum 2 (sites outside of Ontario, Canada) began implementation in September 2020. Implementation was delayed due to COVID-19, particularly with the Stratum 2 intervention group to ensure program delivery was safe to proceed and aligned with local public health guidelines. In brief, Hockey FIT was designed as a lifestyle intervention for hockey team fans who identified as a man, between 35 and 65 years of age, with a body mass index (BMI) of at least 27 kg/m^2^, and deemed safe to exercise via screening questionnaire or health care provider approval. The intervention consisted of a 3-month active phase followed by a 9-month minimally supported phase. The active phase included 12 weekly, 90-minute, off-ice sessions including: (i) group-based exercise consisting of aerobic, body weight, and strength training components and (ii) classroom-based education focused on behaviour change strategies, physical activity and healthy eating. Participants also had access to the Hockey FIT Locker Room App which had features to track steps, compete against other teams’ step counts, and communicate with other participants and coaches.

A total of 42 sites (39 in Canada and 3 in USA) were included on the basis of partnerships with a local major junior or professional hockey team and a local implementation partner (i.e. health/fitness facility, university kinesiology or health-related department, or city recreation centre). Personnel from local implementation partners were trained by the research team as Hockey FIT coaches through eLearning modules, and attending a 2-day in-person or virtual synchronous training session. Affiliated hockey team personnel (e.g. players, coaches) were encouraged to attend sessions as guest speakers to further engage with their fans during the intervention. Hockey FIT participants were recruited via hockey team social media posts and email blasts to season ticket holders, community posters, and word of mouth.

A process evaluation was conducted with sites allocated to the intervention group (*n* = 20) to explore the implementation of Hockey FIT beyond the pilot. Exploring implementation within local contexts aids in the sustainability of interventions beyond the trial time period, and informs adaptation of the intervention to new contexts [32]. Acceptability was deemed critical to examine as there is a greater likelihood of participant and provider adherence to program components and better outcomes if an intervention is deemed acceptable [32]. Acceptability can be defined as the ‘extent to which people delivering or receiving a health care intervention consider it to be appropriate’ (Sekhon, Cartwright, and Francis [32], p. 4). When evaluating acceptability, there must also be consideration of how the intervention can be replicated [34]. In exploring replicability, we looked at the adaptations that were made and suggested changes by coaches to improve future implementation.

### Data collection

#### Focus groups with participants

Hockey FIT participants (referred herein as participants) were recognized as ‘completers’ (i.e. attended ≥50% of active phase sessions including a session in the final 6 weeks) or ‘non-completers’ (did not attend at least 50% of active phase sessions). Overall participant demographics have been described elsewhere [30, 31]. Completers from intervention sites were invited via email to participate in an audio recorded, semi-structured, Zoom focus group (FG) with the research team at the end of the active phase. In the intervention group, 61% (302 of 497) of participants were completers attending an average of seven sessions and were invited to participate in an FG if they previously consented to participating in a FG and audio recording [31]. The research team allocated participants across sites to FGs to capture a variety of experiences across implementation sites. Seven FGs and one interview were conducted (see **Table 1** for attendance).

**Table 1:** Focus group attendance

FG #	1	2	3	4	5	6	7	8
*n*	1^a^	4	6	5	3	4	6	6

^a^More attendees were scheduled but did not show or cancelled last minute.

FGs and the interview lasted between 50 and 60 minutes. One FG turned into an interview after three participants did not show or cancelled on the day of. We included the interview as a data source given interviews can still assist with contextualizing findings from FGs, allowing for a richer description of the participants’ experience [35]. Data collection was facilitated by a skilled moderator, as well as an assistant moderator who took field notes. The moderator used a semi-structured interview guide (see **Table 2** for questions). Participants were asked to verbally consent in addition to written (digital) consent prior to audio recording of the FG.

**Table 2:** Focus group and interview questions

Participant focus group questions
3. What are the reasons you joined Hockey FIT? 4. What were your expectations when you joined? 5. What did you like best about the program? 6. Which program components did you feel were less impactful? 7. What changes would you recommend? 8. How did you feel about the hockey team’s involvement in Hockey FIT? 9. What’s the most important thing that you think the Hockey FIT research team needs to know about your experience?
Coach interview questions
10. What are your thoughts about how the program went overall? 11. What contributed to your level of preparedness when delivering Hockey FIT? 12. In which areas, if any, did you feel you could have used more support? 13. Which components and/or sessions do you think were the *most* effective in helping the men change their lifestyles? 14. Which components and/or sessions do you think were *least* effective in helping the men change their lifestyles? 15. During program delivery, what surprised you? 16. What, if any, adjustments did you need to make when delivering the Hockey FIT program? What led you to have to make these adjustments? 17. What was your most memorable moment or session of Hockey FIT? 18. How did you feel about the Hockey teams involvement in Hockey FIT? 19. What changes, if any, do you recommend to improve the Hockey Fans In Training program? 20. Would you deliver Hockey FIT again?
Implementation partner interview questions
21. What were the reason(s) your organization decided to participate in Hockey FIT? 22. What was your overall impression of the Hockey FIT Program? 23. What did you see as the role of your organization within the Hockey FIT program? 24. In what ways did you feel that your organization was able to appropriately support the program? 25. What, if any, gaps did you experience in terms of your knowledge or understanding about the: a) purpose of the Hockey FIT Project; and b) status of the project activities. 26. What surprised you about your involvement in Hockey FIT? 27. What impact, if any, do you feel the Hockey FIT program had on your organization? 28. To what extent do you see Hockey FIT as a sustainable program for your organization? 29. What would you recommend to make the Hockey FIT program more sustainable for your organization?

#### Participant feedback questionnaires

Participants from intervention sites were contacted via email after they finished the active phase and asked to complete an online feedback questionnaire. Participants (*n* = 316) were asked open-ended questions about: (i) their overall experience with the Hockey FIT program (e.g. classroom content, physical activities, coaches, etc.); (ii) their experience with research-related aspects of the study (e.g. assessments, questionnaires, communications, etc.); and (iii) additional comments they wanted to provide.

#### Interviews with coaches and community partners

Intervention site coaches (*n* = 46) and implementation partners (*n* = 2 per site) were invited via email by the research team to participate in a one-on-one semi-structured interview over the telephone or via Zoom. Interviews with coaches (*n* = 22) lasted between 23 and 60 minutes and were audio recorded upon consent. Interviews with partners (*n* = 21) lasted between 12 and 28 minutes (see **Table 2** for questions).

#### Post-session coach reflections

Coaches from the intervention sites were asked to complete an online questionnaire through Qualtrics across the 12 weeks of implementation. Coaches were asked to comment on a specific set of key tasks to be delivered during each session as per the coach handbook and indicate adaptations they made during delivery. These self-reported reflections were completed weekly, with coaches describing how session delivery went to contextualize program fidelity, adaptations, and suggestions for improving the program.

### Data analysis

Recordings were transcribed verbatim by a third party, and identifiers (e.g. names, locations, dates) were removed prior to analysis. Analysis was conducted through a process of deductive content analysis by question [36]. Steps were taken to ensure data trustworthiness [37]. After each response to a question, the response was provided back to the program participant(s), implementation partner, or coach confirming the response was accurately understood (credibility). At least three members of the research team independently read through and analysed the transcripts on their own (confirmability). Independent findings were discussed, compared, and a consensus was reached on main themes from the responses (dependability). Exemplar quotes for each theme were identified, circulated, and reviewed by the group. Any disagreements were discussed further, and a final list of themes related to participant, coach, and partner experiences were summarized [38].

Data saturation pertaining to the question asked was reached within the responses when no new themes presented in subsequent analysis of new data. To enhance rigor and trustworthiness of researcher interpretation, the research team triangulated data from participants (i.e. FGs and program feedback questionnaire), coaches (i.e. post-session reflections and interviews), as well as implementation partners to confirm themes across data collected [33].

## Results

Three main themes spoke to the acceptability of the program and fidelity and adaptations to inform further program improvement: (i) Motivations for Joining Hockey FIT (group similarities, intrinsic and extrinsic health benefits, hockey team connection); (ii) Effective Program Components (relationship with coaches and other participants, nutrition and exercise); and (iii) Adaptations & Suggestions for Improvement (timing, retention, stronger connection to hockey, Hockey FIT App, use of older version of Canada’s food guide, COVID-19). Participant characteristics (i.e. age, ethnicity, education, and BMI) did not differ extensively between completers and non-completers except for marital status (see **Table 3** for details). Exemplar quotes are included from participants (P), coaches (C), and partners (IP).

**Table 3:** Completer versus non-completer characteristics (intervention group only)

Characteristic	Completers (*N* = 302)	Non-completers (*N* = 195)
Age in years, mean (SD)	50.3 (8.2)	46.7 (7.3)
White ethnicity, *n* (%)	287 (95)	159 (89)
> High school education, *n* (%)	297 (99)	176 (98)
Legally married, *n* (%)	250 (83)	126 (70)
Body mass index (kg/m^2^), mean (SD)	34.3 (5.4)	36.1 (7.1)

### Program acceptability

#### Motivations for Joining Hockey FIT

Reasons participants joined the program included: (i) Hockey FIT being a program that targeted men of similar size, shape, goals, and interests; (ii) to attend to health intrinsically (for personal reasons) and/or extrinsically (for family/friends); and (iii) team connection. As noted by one participant: ‘It wasn’t just, “Hi, I’m just some *guy* offering my workout program”. That it’s tied in with the [hockey team], whichever team that might be, it has some legitimacy’ (P08). Program coaches saw Hockey FIT as targeting a unique group who may not feel comfortable attending traditional fitness classes. One coach commented:

It’s just really targeting that group of guys that aren’t necessarily coming in for a spin class and get them into something they can do together, and they feel comfortable, they can chirp each other a little bit and talk about hockey and talk about stuff (C03).

Intrinsically, participants realized they were not as active as they once were. As reported by a participant:

I was active twenty, thirty years ago and I had a job that consisted of a lot of physical work and lately I’ve been more tied to the office and I am hitting early sixties, so realizing that I was becoming a couch potato (P07).

Additionally, participants wanted to improve their health for themselves and their family. As one participant noted: ‘I wanted to lose weight and be healthier for myself, for my kids, for medical issues that pop up because it’s all tied to weight’ (P09).

#### Effective Program Components

The term ‘effective’ resonated with participants and coaches as components which informally through the group dynamic (i.e. relationships built with coaches and other participants) and formally through program content (i.e. nutrition and exercise content) supported behaviour change. Coaches reiterated how successful they thought the program was:

A lot of [participants] were seeing the weight loss that they were hoping to see throughout the program and we actually made some pretty good strides forward not just with their dietary concerns, but the mentality with it as well (C06).

Partners found the program was easy to implement as the expectations of involvement were laid out ahead of time. Participation provided partners with positive branding by engaging more with their fans. As noted by a partner: ‘It really helps with the message that we want to have out there and that we work with local partners and national partners, regional partners and just what the [Hockey Team] platform can be besides just watching hockey games’ (IP15).

##### Relationship with program coaches

Participants emphasized their positive experiences with their coaches. As a participant stated: ‘[The Coaches] were engaging, they were personable. You didn’t feel intimidated by them, and they were excellent at their job and helping us figure [program concepts] out’ (P05). Coaches reported how important it was to build relationships with participants. As one coach described: ‘They’re not gonna come to a program if they think I’m a jerk or they don’t believe in what I’m trying to provide for them’ (C10). In addition, participants found the skills the coaches had in fitness were beneficial in tailoring program components to participant needs. One participant stated: ‘Some of the guys couldn’t do a bending exercise or couldn’t do a running exercise and our trainers would offer alternative exercises which we could then take home and continue to do’ (P13). Coaches reiterated their background and life experiences played a role in feeling prepared and confident delivering content. Coaches also found the resources provided by the research team (i.e., handbook, training workshop, and eLearning) helped them feel prepared and ready to deliver the program again. Partners saw benefit in having their own staff deliver Hockey FIT. One partner stated:

We were able to train our own staff and have our own staff deliver the program. I think that was an important part because the program still would have been successful with outside instructors, but just us being able to use our own staff made it even more effective (IP03).

Partners expressed appreciation for the well-developed and organized program, underscoring these attributes as key for the ease with which they could deliver the program.

##### Relationship with other participants

The group dynamic throughout the 12 program sessions was recognized across participants, coaches, and partners as creating a strong foundation for being open about successes and struggles, building in accountability for attending sessions, and showing up to support each other in making behaviour change. As noted by a participant: ‘I also enjoyed that there’s a group of guys sitting there waiting that are going to be there, so it forced me to say, “yeah I gotta go with them because they’re going to be waiting for me”’ (P27). Additionally, participants found the program provided a safe environment to share personal experiences and relate to others’ struggles. One participant described this as, ‘…it’s the no judgement, we’re all here, we’re all adults, we’re just trying to lead a healthier life and learn. And just that attitude and group support is what makes [Hockey FIT] successful’ (P02). Coaches noted cohesiveness across the group where they could brainstorm ideas of how to achieve the goals they set in the program. One coach stated: ‘…it was their drive and their ability to support one another that really kind of drove their success’ (C10). Participants liked how the coaches celebrated their success during the program. One coach noted how they celebrated successes by asking participants to, ‘give me a win of how your week went’ (C03). Participants expected this routine as the program progressed, leading this activity themselves if the coach forgot seeing it as a key part of monitoring progress. Partners were surprised by how open and welcoming the participants were and friendships that were created. A partner noted this by saying:

When everyone was getting introduced to one another, you can tell there were some hesitations, apprehension around the table. Guys didn’t necessarily know each other, but by the end of the 12 week sessions, it was incredible to see friendships forged (IP06).

##### Nutrition and exercise

In exploring the perceived effectiveness of program components that helped to create behaviour change, both participants and coaches noted the benefits of the classroom-based nutrition content. Participants found the nutrition components (i.e. food labels and plastic food models) were helpful tools to improve their diet. Coaches expressed that the nutrition components resulted in many ‘aha’ moments for participants in having them see what a recommended serving and portion size was compared to what they would normally eat. The models were effective in creating these moments. As one coach said:

When you start handing out the meat products guys were sitting there looking at them and they were like this is not at all what I intake at home on a daily basis. I think this was the first time that I ever saw them open their eyes to the reality of things (C06).

Along with the nutrition components, the exercise session was also noted as effective by program participants and coaches for its simplicity. Participants liked that they were simple to do with one participant saying: ‘we weren’t necessarily using the big fancy machines, we were doing stuff that you can do with your body weight and a wall and maybe a chair’ (P07).

### Fidelity and adaptations

For the post-session coach reflections, 81% of sites completed these for each week of program delivery with 93% of coaches indicating implementation of key tasks went well. There were 18 out of 240 instances of a site not completing a post-session coach reflection during the program (1 site in session 7; 3 sites in session 8; 3 sites in session 9; 2 sites in session 10; 4 sites in session 11; and 5 in session 12).

#### Adaptations & Suggestions for Improvement

Even though coaches reported the delivery of most key tasks went well, there were adaptations and suggestions made to improve the program. These subthemes related to: (i) timing, (ii) retention, (iii) a stronger hockey connection, (iv) Hockey FIT App, (v) use of an older version of Canada’s food guide, and (vi) COVID-19.

##### Timing

Coaches reported keeping on time was a challenge, especially with guest speakers or when the group was highly engaged in discussion. As noted by a coach:

I’m going to say timing on the workouts. We weren’t going to shut them down when we were having discussion, people were asking questions, so there was a couple of times the fellas started opening up and asking questions, then come the workout times. Our group was consistently running late (C07).

A suggested improvement was to meet a bit earlier these days to allow for flexibility.

##### Retention:

Coaches were surprised by the retention challenges, although noted it may not have been caused by the program itself. Hosting the program in the summer, work commitments, or injuries were noted to explain some absences. There were also unique challenges such as COVID-19 public health restrictions with Stratum 2. Coaches noted having to adapt activities to create smaller groups or less stations in the exercise sessions pending attendance. Suggestions were focused on increasing competition to build comradery and accountability across the group by including more hockey-related activities. Additionally, a suggestion was to identify a peer team captain to encourage attendance and support those struggling with commitment.

##### Stronger connection to hockey

Participants and coaches felt there could have been a stronger hockey connection and team participation in the program was not consistent across all sites. As noted by a coach: ‘It would’ve been cool if we had the first orientation session and if it started off with a ball hockey game and maybe one of the players had shown up and participated’ (C06). Coaches and partners mentioned the summer and playoff season, in which the program did run for some sites, impacted the team’s availability. When the hockey teams were engaged in the program, the participants and coaches expressed appreciation for the opportunity for some inside knowledge of the team. Engaged hockey teams also desired to be present in the program to show their appreciation to their fans. A partner reported being: ‘A welcoming force to these guys. Like “Hey, you guys are [team] fans, we really appreciate all the support you show us. Here is how we can give back to you guys and make your lives better”’ (IP06).

##### Hockey FIT App

The Hockey FIT Locker Room App was an area in which both coaches and participants desired changes, specifically the step tracking. As reported by a participant, ‘…it didn’t seem to be in sync with the internal steps that the phone already calculates by…’ (P13). Coaches also noted the app could have been more user-friendly and focused on more than step counts for activity tracking. Integrating wearable devices (e.g. FitBit^©^) was identified by coaches to address the concerns with accuracy in tracking steps.

##### More exercise

Participants expressed a desire to have more exercise earlier on in the program, allowing for a more gradual increase in intensity. A participant stated, ‘I understand that starting off slow is important just to see where everybody is at. After that, there were days after the workout at the [community fitness facility], it took three days to recover’ (P09). Coaches reported adjusting exercises for participants to ensure they were being safe, but they also desired more opportunities to integrate exercise earlier in the program to allow for a gradual progression.

##### Use of older version of Canada’s Food Guide

Coaches and participants noted issues using the 2007 version of Canada’s Food Guide when a new version was released in January 2019 [39]. This was thought to drive some participants away from the program. As noted by a coach: ‘A lot of the people were put off by the fact that we were using an older version of the Canada’s Food Guide. They were just saying that “I’m not realistically going to use this food guide”’ (NC09).

##### COVID-19 adjustments

The pandemic impacted various aspects of group dynamics, comradery, and setbacks faced by participants. Some participants reported a loss of comradery in the group due to fear of getting close to others and increasing likelihood of viral spread, as well as pandemic restrictions at gyms and fitness facilities that led to program-specific adaptions (e.g. spaced out activities). Coaches noted COVID-19 resulted in a lack of hockey team engagement. A small number of sites were required to complete several sessions online via Zoom, with coaches reporting this impacted group participation. Despite these challenges, COVID-19 was also a motivator for some participants to join the program.

## Discussion

Exploring acceptability and areas for adapting the program is important to inform future program iterations. In relating to the proposed research objectives, the extent to which participants, coaches, and partners viewed Hockey FIT as acceptable for encouraging health behaviour change was impacted by various factors. As demonstrated through this evaluation, Hockey FIT is acceptable at recruiting middle-aged men into health promotion programs and the group dynamic between participants and with coaches fostered a supportive space to encourage and make health behaviour changes. Further, coaches and partners found Hockey FIT was easy to deliver although there are areas requiring further attention to improve program acceptability.

Many interventions have focused on engaging men through group-based activities geared towards those with similar attitudes and behaviours towards health [14]. A finding from this study was that participants, coaches, and implementation partners viewed hockey fanship as an acceptable motivator to address men’s health. This theme further speaks to the importance of bringing together individuals with similar interest as it can build a group dynamic which supports behaviour change. Group dynamic theory posits individuals can be attracted to a group’s task function (i.e. activities of mutual interest), as well as social function (i.e. connecting with community members) [40]. This theory can be used to explain how motivations for joining Hockey FIT was to improve their health (task function) as well as the opportunity to connect with other hockey fans of similar size, shape, and location (social function). Other studies, including the Hockey FIT pilot study, found similar success suggesting main drivers for engagement of difficult to reach groups should combine task and social functions [15, 16, 23].

Retention was an issue identified by participants and coaches. Research has supported targeting ‘men’ friendly community environments is key for recruitment, but retention requires sustaining community in these spaces [14]. Retention requires continuing to address what is desired from a health promotion program for men of which this evaluation sheds further light on. The findings from this study do support that hockey fanship can be a significant draw for men looking to make health behaviour changes. During program, participants highly valued the resulting comradery, desiring more competition to increase these connections across the group as these factors helped them to achieve their goals. As noted by Oliffe (2020), collaborative leadership models, whereby leadership is shared across a group and encouraged across peers in the program not resting solely with program administrators, can improve not only retention within program, but leads to commitment and recruitment when/if the program runs again [41]. Of note, retention rates with this study were still on par with similar studies targeting sports fans even though they were noted as an issue by participants and coaches [42].

The global COVID-19 pandemic impacted Stratum 2 as implementation had to be halted and adapted to limit exposure and align with health guidelines. As noted by Barroga and Matanguihan (2020) [43], there were fundamental shifts in research processes during the early years of the pandemic, impacting continuity. Further, COVID-19 required rapid adaptation from in-person to virtual, impacting intervention delivery and experiences with delivery. Research with group fitness instructors during COVID-19 found online teaching was more demanding and there were difficulties building rapport [44]. A limitation to this research could be not explicitly asking how the pandemic impacted the delivery of the Hockey FIT program to provide a comparison to pre-pandemic delivery.

Further limitations include participant data only being included from intervention sites. Therefore, this study would not reflect implementation at all sites. Participant data were only collected from individuals who completed the program, given that these individuals would be able to best reflect on their experience with the program. The perspectives of individuals who withdrew would provide further information on intervention improvements to meet participant needs and increase retention. Further, findings may be transferable to other health promotion programs using sport fanship in North America, but this study primarily recruited white men who had a greater than high school education, and therefore, would not be representative of all fans. There were practical and logistical challenges with having a third party objectively measure program fidelity across intervention sites. As fidelity was measured through self-report from coaches, the findings speak more towards the experience of implementing Hockey FIT as designed rather than objectively whether the program was delivered as intended.

## Conclusions

Key takeaways from this study include engaging communities with similar interests (e.g. sport) as a valuable outlet for promoting health behaviour change with harder to reach groups. Implementation should capitalize on group dynamics and activities that facilitate greater social connection with participants to reduce retention loss. Sports represent an effective way to engage men in health promotion interventions, but there are groups with whom sport may not be of interest and yet are interested in making lifestyle changes [45]. In replicating these findings, identifying groups or bringing together individuals with common interests could be a strong foundation for behaviour change. Therefore, the scalability of Hockey FIT to other sports, activities, and demographics should be further explored to potentially reduce rates of chronic disease in many communities.

## Data Availability

De-identified data from this study are not available in a public archive. De-identified data from this study will be made available (as allowable according to institutional IRB standards) by emailing the corresponding author.
